# Magnetic Resonance Imaging with Active Implantable Hearing Devices: Reports from the Daily Radiological Routine in an Outpatient MR Center

**DOI:** 10.3390/jpm13081220

**Published:** 2023-07-31

**Authors:** Julia Fruehwald-Pallamar, Franz Fruehwald, Laura Holzer-Fruehwald, Richard Nolz, Christian Stoiber, Georg Mathias Sprinzl

**Affiliations:** 1 Institut Fruehwald und Partner, 3100 St. Poelten, Austria; 2 Department of Otorhinolaryngology, Head & Neck Surgery, University Clinic St. Poelten, 3100 St. Poelten, Austria; 3 Karl Landsteiner Institute of Implantable Hearing Devices, 3100 St. Poelten, Austria

**Keywords:** hearing implants, magnetic resonance imaging, dislocation, safety policies

## Abstract

Purpose: For people with hearing implants (HI), magnetic resonance imaging (MRI) still presents some difficulties due to the built-in magnet. Radiologists often have concerns regarding complications associated with HIs. The aim of this study was to record the experiences of HI users during and after MRI examinations. Method: A survey including 15 questions regarding MRI specifics, namely changes in hearing ability, hearing/sound impressions, pain, uncomfortable feelings, etc., were mailed to our patients. Results: Overall, 79 patients with HI had a total of 159 MR examinations in our institute. A total of 45 HI recipients reported back: 35% stated that they had been rejected by an MRI Institute because of their HI. Their feelings/impression ratings during the measurements were not present and therefore were not rated for the majority (49%), 42% of the HI users rated the pain with 0 (no pain), 2% with 1 (very light pain), 4% with 5 (acceptable pain), and 2% rated the pain with 7, which is between acceptable and strong pain. One examination resulted in a dislocation of the magnet of a cochlear implant (CI 512 Cochlear Limited). No adverse events were reported for MED-EL HI users in the survey (none of the contacted AB users answered the questionnaire). The reported mean daily wearing time was 11.6 ± 4.6 h per day for 6.3 ± 1.7 days per week. Conclusions: Based on these results and our experience we conclude that MRI examinations with HI are safe given that the measurements are performed according to the safety policies and procedures released by the manufacturers.

## 1. Introduction

The increasing diagnostic importance and the ever more frequent use of MRI devices have led to the fact that, according to statistics, every person needs an MRI scan at least once in their lifetime. From diagnosing a disease to monitoring the success of therapy, MRI has become an indispensable part of modern medicine. It is expected that three out of four people will need an MRI scan in the next 10 years [1]. An MRI scanner consists of a large magnetic coil generating a strong and constant magnetic field whose strength varies from one scanner to another. It ranges from 0.2 Tesla (T) to 7.0 T or higher. Most radiology departments around the world use 1.5 T or 3.0 T MRI scanners [2,3,4]. Due to the strong magnetic field which interferes with the magnet of the HI users devices, radiologists often have concerns performing MRI scans since complications such as pain, magnet displacement, depolarization, and polarity reversal have been documented in the past. Additionally, for diagnostic purposes of areas close to the implant site, artifacts caused by the implant remain an issue but may be reduced by the application of specific metal artifact sequences. The cause of these possible complications is the magnet built into the implant. With some HI, such as the cochlear implant (CI), the magnet can be partially or completely detached from its housing by the forces acting on it in the MRI. This causes pain and requires surgical intervention to bring the implant magnet back into the correct position [5,6,7,8,9,10,11]. For this reason, manufacturers of CIs developed diametrical magnets that could rotate freely in their housing to compensate for the forces of a 3.0 T magnetic field (e.g., post-1994 generations of CI’s from MED-EL) [10,12]. Depending on the model, different safety precautions or scan parameters must be observed during scans with HI’s, for example the survey revealed two CIs from Oticon, the model of the implant was the Neuro Zti, which is according to the manufacturer policies and procedures MR conditional at 1.5 and 3.0 T with the implant magnet in place [13,14]. The standard for one of the survey participants is similar, who has a Cochlear Nucleus CI512 implant, which is approved for MR scans under specific conditions at 1.5 T with the magnet in place and at 3.0 T with the magnet removed.

A literature review on MRI with HI resulted in 34 studies, out of which 17 papers included implants from Cochlear Limited, 13 reported on implants from MED-EL, and 8 on devices from Advanced Bionics (AB) [5,6,7,9,15,16,17,18,19,20,21,22,23,24,25,26,27,28]. The total number of patients of the included studies was 440 out of which 215 patients underwent 368 scans in total. Of the 215 patients with MRIs done, 82 were from MED-EL, 70 from Cochlear Limited, 22 from AB, and 4 from Oticon Medical. In total, 24 studies reported the use of head bandages in preparation for 228 MRI scans; no head bandages were used in 81 scans. In preparation for the imaging exams, 25 magnets were surgically explanted and 29 scans were conducted without a magnet in situ. In total, 41 patients were either under anesthesia or sedation for the duration of the scanning. Out of the investigated manufacturers, 17.3% (19/110) of MED-EL patients, 19.8% (23/116) of Cochlear Limited patients, and 62% (21/31) of AB implantees reported pain during an imaging episode. In total, 21 scans had to be aborted before finishing the examination: 18 due to pain (4 MED-EL, 9 Cochlear Limited, 3 AB), 2 due to anxiety (1 Cochlear Limited and 1 AB), and 1 due to artifacts (MED-EL). In 34 cases, a complication with the implant magnet occurred during or after the MRI scan. Specifically, 30 of these complications were dislocations (30 Cochlear Limited., 6 AB, 1 MED-EL middle ear implant, 3 unknown manufacturers). The scanned middle ear implant generation VORP 502 was not MRI conditional and should not have undergone an MRI examination. None of the reviewed studies with the MED-EL Bonebridge device reported pain, discomfort, or abortion of the scans [5,6,7,9,15,16,17,18,19,20,21,22,23,24,25,26,27,28].

Hence, manufacturer recommendations (summarized in Table 1) should be followed to reduce the risk of complications, although complications may occur even when guidelines are followed. For this reason, at our radiologic institution including MRI possibilities, the indication for imaging these patients is always thoroughly reviewed and patients are counseled before giving individual consent. Therefore, the aim of this observational survey study was to record the experiences of our patients with HIs during and after routine MRI examinations.

## 2. Materials and Methods

**Figure 1:**
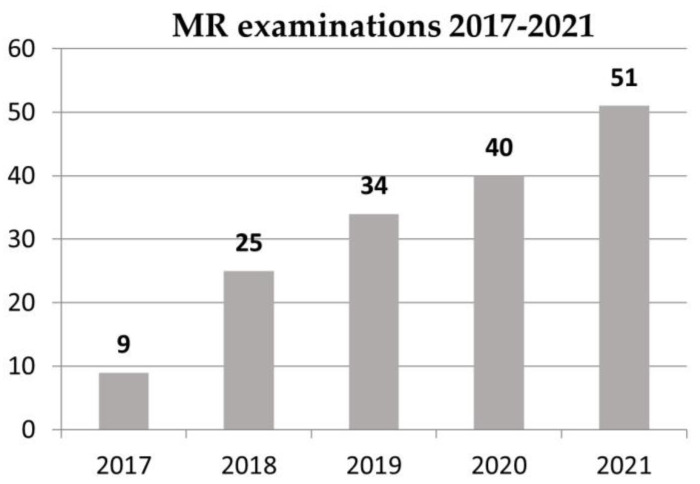
MRI examinations of implant users between 2017–2021.

Between January 2017 and December 2021, we performed 159 MR examinations on 79 patients with hearing implants in our outpatient MR institution (Figure 1).

For 13 patients, 40 examinations were performed on bilaterally implanted patients. In total, 38 patients underwent more than one MR examination. In total, 3 patients (MED-EL synchrony) were scanned on 3.0 T, all other patients were scanned on 1.5 T. The majority of patients had a spine or knee scan (figure MR regions).

A survey including 15 questions about MRI experiences with HI was developed. The survey retrospectively collected information relating to the implant type and the date of implantation, the daily wearing time of the audio processor, whether the subjects were rejected by an MRI institute, and whether investigations have taken place in other institutions. The survey obtained the magnetic strength of the MRI, the date of the MRI procedure, how many times an MRI was performed, the examined region, the perceptions during the examination, and the need for MRI interruption. Data were collected concerning possible complications including hearing impressions, magnet strength, and eventual consequences like revision surgeries.

**Figure 2:**
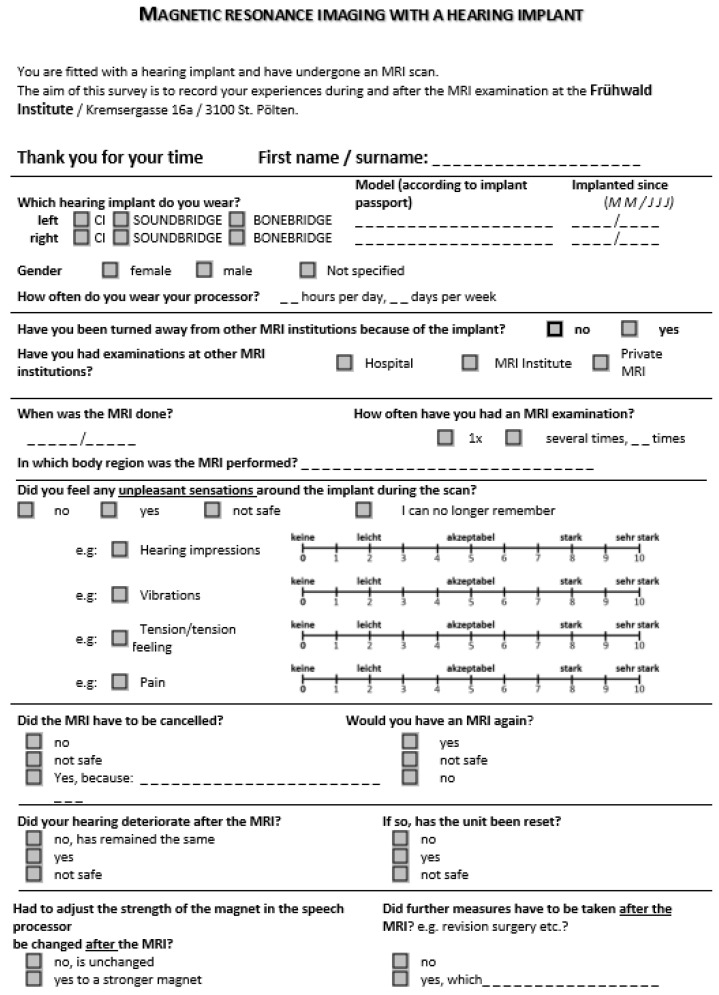
Translated questionnaire. The questionnaire used for the study was distributed in German, the native language of the patients.

Patient specifics included all subjects who were implanted with an active implantable hearing device, already scheduled, or who had undergone an MRI measurement for routine diagnostics of any part of the body in the institute. Subjects who already had undergone an MRI examination received the questionnaire by mail and subjects scheduled for examinations received the questionnaire after their appointment (Figure 2). The survey collected anonymized data and information. The participants completed the survey offline and returned it via email, mail, or in person. The study duration was 6 months. In total, 79 questionnaires including 92 implants were distributed. Ethical approval was obtained from the Institutional Review Board for Lower Austria, Office of the Lower Austrian Provincial Government (approval number: GS1-EK-4/711-2021).

## 3. Results

In total, 159 MR examination on 79 patients were successfully performed on 1.5 T (156) or 3.0 T (3 patients). In this time period, 4 patients could not be scanned due to subjective discomfort when entering the 1.5 T MR gantry (Pulsar Ci100, Mi1000 Concerto 2x, BCI 601, (MED-EL GmbH (6020 Innsbruck, Austria)); in those cases, the scanner was not started.

From the 79 distributed questionnaires, 45 patients completed and returned the questionnaire. The evaluated data include 53 ears.

### 3.1. Distribution of Implant Manufacturer and Implant Types

**Figure 3:**
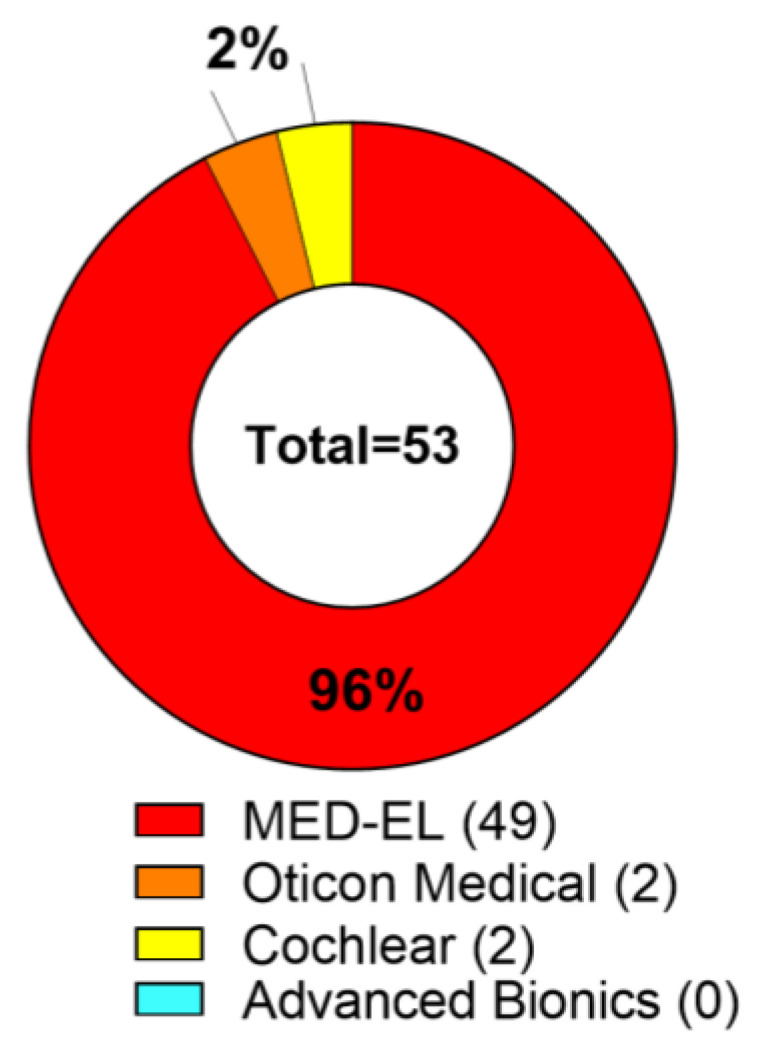
Distribution of implant manufacturer.

**Figure 4:**
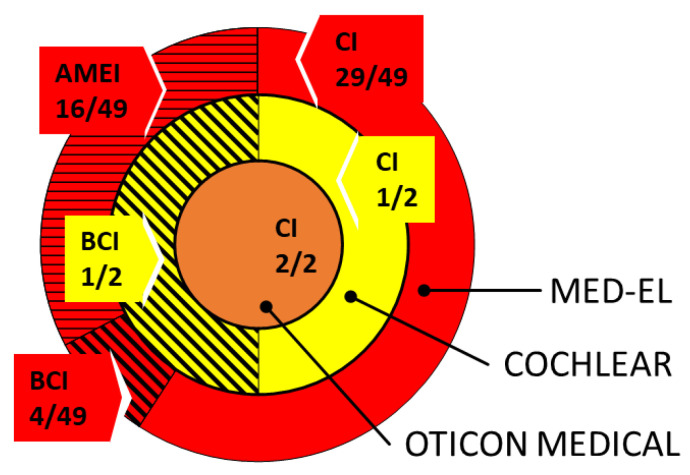
Distribution of HI among the manufacturers. (Company colors are as follows: red: MED-EL; yellow: Cochlear; orange: Oticon).

The questionnaire return rate was 58% which means 43 implant users (53 ears) out of the 79 returned the survey. Calculations from now on deal with implant ears not subject numbers. The majority of the responses involve 96% MED-EL (6020 Innsbruck, Austria) implant users: 2% are fitted with an implant of Cochlear Limited (2000 Sydney, NSW, Australia) and 2% use an Oticon Medical implant (2765 Smørum, Copenhagen, Denmark), no users from the implant manufacturer Advanced Bionics (91355 Valencia, California, United States) responded. The described distribution of manufacturers is shown in Figure 3. Out of the total population, 60% of the participants were implanted with a cochlear implant (CI), 30% were fitted with an active middle ear implant (MEI), and 10% use a bone conduction hearing implant (BCI). In total, 91% of the CI users were implanted with a MED-EL device, 6% with Oticon Medical, and 1% with a CI of Cochlear Ltd. All MEIs were from MED-EL. BCI were in 80% from MED-EL and 20% from Cochlear Limited (Figure 4, absolute data distribution).

### 3.2. Demographic Data

The patient demographic data are summarized in Table 1. Out of the 32 CI implanted patients, 13 were female and 19 were male. In total, 16 patients (9 female and 7 male) using an MEI and 5 patients with a BCI (3 Bonebridges and 1 Baha) were included in the study. The mean age at examination of CI users was 63.5 years (range: 36–82 years). MEI users were 66.38 years on average (range: 51–84 years) and the mean age of BCI patients is 55.25 years (range: 45–73 years). The CI users stated their mean daily wearing time as 13.24 ± 3.58 h (range: 4–18 h). The MEI implant users reported a daily mean audio processor wearing time of 8.83 ± 4.18 h (range: 3–16 h). A mean daily wearing time of 9.5 ± 2.87 h (range: 5–12 h) was reported by the BCI users. A total of 94 MRI examinations concerning these HI patients were performed (Table 1).

In the study, 38% of CI users, 38% of MEI users, and 50% of BCI users stated that they have been rejected by an MRI Institute because of their HI.

### 3.3. Examined Body Regions

**Figure 5:**
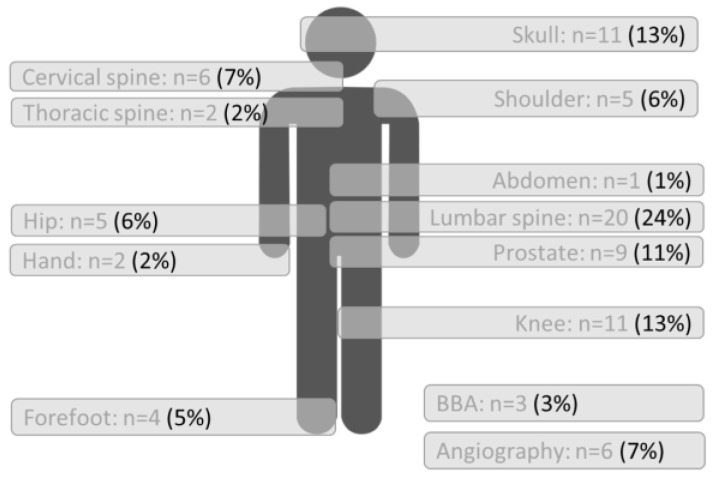
Examined body regions; total number, and percent (%) for all hearing implants.

The questionnaire identified the examined body regions. The distribution of the examined regions of all implant users is shown in Figure 5. The distribution per implant were as follows: most examined body regions of CI users were lumbar spine (33%) and prostate gland (17%). For the MEI users the most examined regions were the skull (26%), knee (19%) and angiography (19%). For BCI users, the most examined region was the lumbar spine (40%) followed by the hip, prostate gland, and knee each with 20%, respectively.

The majority (35%) of the study population had one MRI examination followed by 30% who had at least two; one subject (MEI; Vibrant Soundbridge user, MED-EL, 6020 Innsbruck, Austria) underwent 10 different examinations without any complications or negative impressions during as well as after scanning.

### 3.4. Patient Perceptions during MRI

**Figure 6:**
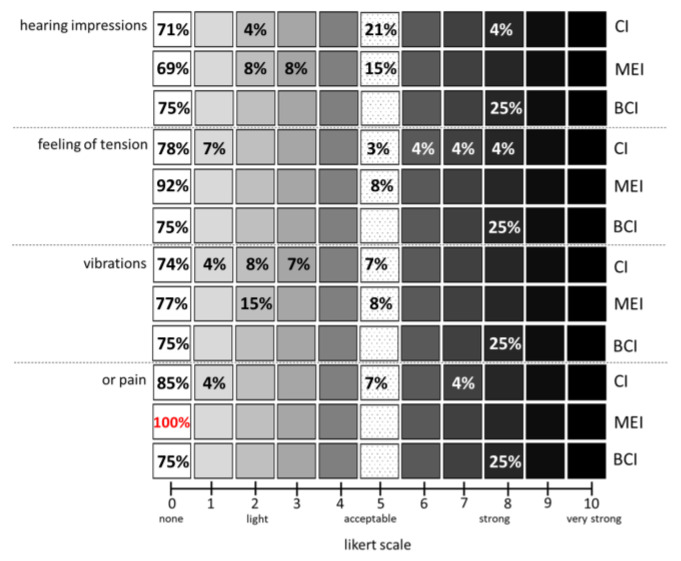
Percentage distribution of different sensations during the measurement (left x-axis) subdivided for CI, MEI, and BCI separately (right x-axis) according to the indicated Likert scale (y-axis). In red highlighted the only implant with no pain (100%).

The questionnaire also examined unpleasant sensations on a Likert scale from 0 to 10 which were then further specified as hearing impressions, feelings of tension, vibrations, or pain the implant users may have experienced during their examinations (Figure 6). Overall, 81% of the CI cohort reported that they experience no sensations during the scans, 4% did not remember, 4% did not answer the question and only 11% answered with yes and further specified the sensations experienced. Acceptable hearing impressions during the scan were reported in 21% of the CI users and 71% rated the hearing impressions with 0. Interestingly, the one subject who experienced a dislocation of the magnet (CI512, Cochlear Corp) rated strong hearing impressions. In total, 85% of participating CI users ranked the pain at 0 on the scale. Overall, 4% of the participants indicated their pain at 1 on the scale and 7% said that the pain was acceptable (5 on the scale). The case of magnet dislocation was a CI 512 manufactured by Cochlear Limited and the patient wore on the contra-lateral side a BCI, a so called Baha from the same manufacturer. This patient therefore also rated in the BCI group and unsurprisingly experienced the hearing impressions, feelings of tension, vibrations, and pain with a 7 (25%, one user out of four). Indeed, his MRI examination was stopped and the implant had to be revised. Overall, 93% of the CI users stated that their hearing did not deteriorate after MRI. One subject (3%) felt that the hearing got worse in relation to CI revision after magnetic dislocation. In total, 4% of the CI user population provided no information regarding hearing deterioration. None of the CI users stated that the magnet strength on the audio processor had to be changed after the MRI and all subjects, except the one dislocation, would undergo the same procedure again. The same applies to the BCI study population indicating no hearing deterioration or need for a magnet change after MRI (except for the contra-lateral dislocation patient).

Overall, 100% of the MEI users ranked their pain at 0 on the described pain scale. The percentage of MEI users who reported the need for a stronger magnet after MRI was 8%.

No pain (0 on the scale) was reported by 75% of BCI users. In total, 25% ranked their pain at 7 on the scale concerning the magnet dislocation of the CI on the other side. This was also the only subject for whom the MRI had to be terminated before the end of the session.

### 3.5. Device Specific Changes Required

The survey also examined whether the MRI measurement and the occurring magnetic field resulted in device specific changes such as a change in implant magnet strength or changes in hearing ability. The CI cohort in 93% of cases reported no changes to their hearing; one subject (the revision subject, CI512 (Cochlear Limited (2000 Sydney, NSW, Australia)) reported a decline in his hearing ability after the examination. None of the CI users reported a change in their magnet strength. In the MEI group (Vibrant Soundbridge, MED-EL, 6020 Innsbruck, Austria), all scans went as planned and 77% reported no change in their hearing ability; none indicated a change but 23% stated that they were not sure if their hearing had changed. One subject received a new fitting after MRI but it is not clear whether that was procedure-related as the question regarding the change in hearing ability was not rated. One subject required a different magnet strength after his examination. The BCI group consisted of four subjects in total, where one was implanted with a Baha (contra-lateral side CI512, both from Cochlear Limited (2000 Sydney, NSW, Australia)). The other three MED-EL Bonebridge users reported no changes in hearing whereas the aforementioned Baha user who had to undergo a revision on the contra-lateral side due to magnet dislocation also required a change in the magnet strength of his Baha device. Except for the one revision subject, all other HI users who answered the question would undergo an MRI examination again (3% and 97%, respectively).

## 4. Discussion

MRI has become an indispensable part of modern medicine for the diagnoses of diseases and/or the monitoring of successful therapy. It is expected that three out of four people will need an MRI scan in the next 10 years [1]. Unfortunately, access to MRI may cause difficulties for people with a hearing implant due to possible complications related to the implant’s in-built magnet. Radiologists often have concerns when dealing with hearing implant users who require an MRI scan, which is coherent with what was reported in this survey. In the study, 38% of CI users, 38% of MEI users, and 50% of BCI users were rejected by an MRI Institute because of their HI.

An MRI scanner has a large magnetic coil that generates a strong and constant magnetic field. The strength of this magnetic field varies from one scanner to another, ranging from 0.2 T to 7.0 T or higher. This strong magnetic field can interact with the internal magnet of the HI which may result in some of the internal interactions which may appear quite intense for the HI user or even cause complications. Eddy currents in conductive elements of the hearing implant could result in the heating of the surrounding tissue. Induced voltages in conductive loops resulting from rf fields can damage the implant. Force and torque on ferromagnetic parts of the implant can move implanted parts and thus harm the patient. The internal magnet’s comparatively strong field can cause artifacts in the MR image. Furthermore, the magnet can be partially demagnetized, resulting in reduced implant functionality and causing pain on the one hand and may require surgical intervention on the other hand. All current HI manufacturers aimed to address those issues in their implant/magnet design and hence, the latest implant generations are between 1.5 T and up to 3.0 T conditional (depending on the manufacturer and type of implant and under specified conditions) (Table 2). The herein-presented survey therefore aimed to investigate HI recipients who had already undergone MRI examinations at our institute regarding the above-mentioned complications and details such as the magnetic strength of the MRI, the date of MRI procedure, how many times an MRI was performed, the examined region, the perceptions during the examination, and the need for MRI interruption were considered. Data were collected concerning possible complications including hearing impressions, magnet strength, and eventual consequences like revision surgeries. The survey was mailed out and the response rate was relatively high with 58% resulting in information from 45 HI recipients including 53 ears.

The literature reports on unwanted complications during MRI scans for CI’s, auditory brainstem implants (ABIs), MEI’s, or BCI’s. These complications include large artifacts or distortions on the MR image, distortion, or dislocation of the implant magnet, weakening of the implant magnet, demagnetization, or an increase in temperature in the vicinity of the implant, dependent on the area of interest/measurement [11].

The presented study revealed that the most examined areas were the lumbar spine, the prostate gland, the skull, and the knee. This is sort of coherent with the results of the literature review, where the most examined body regions are the head and the spine [11]. The most common complication is magnet dislocation and in this case the MRI examination has to be interrupted due to pain [5,6,7,8,9,10,11]. The reason for rejections could also be a lack of information of the radiologists about the different safety precautions and scan parameters that must be considered when conducting an MRI scan.

There were no issues with artifacts identified in the study which goes along with the literature review where only one case was described that was dealing with artifacts [11]. In general, artifacts are an issue only if the examination area is very close to the HI, namely in the skull region. Several studies showed that the application of artifact reduction sequencing and an adaptive positioning of the implant enable regular MRI examinations of the region without affecting the diagnostic image quality for diagnostics [30,31,32]. Our survey established that all the MRI scans performed with MED-EL HIs occurred without any magnet dislocation. This confirms the suitability of the diametrical magnets of MED-EL CI and auditory brainstem implant (ABI) HIs in a 3.0 T magnetic field and the approval of the Vibrant Soundbridge VORP 503 and the BCI 602 at 1.5 T magnetic field [12,14].

In the survey, the patients rated their pain between non-existent and acceptable pain (25 MED-EL CI, 2 Oticon medical). There was only one adverse event causing strong pain due to a magnet dislocation (Cochlear Limited CI). Similar magnet dislocation associated with pain concerning the Cochlear Limited CI 512 was described in the literature frequently [5,6,7,9,15,16,17,18,19,20,21,22,23,24,25,26,27,28].

None of the subjects participating in our survey required the surgical removal of the magnet to undergo MRI; hence, no patient had to be under anesthesia or sedation for the duration of the scanning nor had hearing downtime because of surgical magnet removal. This may be due to the fact 92% of MED-EL HI users of the survey cohort pertained to special magnet design not requiring magnet removal; also, children were not part of the study [5,6,7,9,15,16,17,18,19,20,21,22,23,24,25,26,27,28] and usually examinations in children often require sedation or anesthesia to perform a radiological examination [33].

The herein-presented survey revealed no complications concerning MEI and BCI. This goes along with the results of the reviewed literature. Complications concerning MEI described in the literature occurred solely when the magnet strength of the MRI scanners was higher than that recommended by the manufacturer (1.5 T for MEI) [11]. The only complication described with BCI is the occurrence of artifacts which may be successfully improved by artifact reduction sequencing [30,31,32].

The literature reported ‘pain’ during measurement as the most common adverse event during MRI examination for HI users and as a result, examinations were stopped before results could be achieved but without any further MRI-related complications [5,6]. Between 75 and 100% of the subjects of this survey reported no pain, except the one magnet dislocation patient who reported pain for both sides, the CI as well as the contra-lateral Baha side (4% and 25%, respectively). Due to this fact, authors of the study recommend the introduction of a pain inventory to counsel HI users prior to MRI examination to avoid unnecessary aborting of measurements. This may allow patients to have a realistic assessment of pain and may prevent patients’ fear that their implant could be damaged.

A limitation of the study is the fact that most of the survey participants were from one company and that the survey was based on questions, containing subjective information and feelings which might have been too long in the past and could therefore not be recalled anymore. However, it confirms the lack of information of some radiologists regarding HI and MRI examinations as a lot of patients are still rejected by MRI institutes (38% of CI users, 38% of MEI users, and 50% of BCI users were rejected by an MRI institute because of their HI). Our survey found that, especially for MED-EL HI recipients, most of the survey participants did not have any complications, namely no dislocations or weakening of the implant magnet when the MRI was performed while following MED-EL’s safety policies and procedures.

## 5. Conclusions

MRI is nowadays one of the most important diagnostic tools in radiology. While performing an MRI examination, the safety of the HI user is the highest priority for both the radiologist and the patient. Results of this survey found that individuals with devices manufactured by MED-EL can safely undergo an MRI at a field strength according to the manufacturer’s safety policies and procedures [14]. The authors therefore conclude that radiology departments should not hesitate to perform MRI scans on HI users as long as all the necessary information and policies and procedures are followed. Precisely because of the fact that the safety of HI recipients during an MRI scan should be the priority, radiology departments/radiologists should become familiar with the respective policies and guidelines to also enable those patients to be diagnosed conventionally by means of an MRI without delays and/or hassle.

## Figures and Tables

**Table 1:** Patient (ear) demographics.

	CI	MEI	BCI
**Gender**	13 female, 19 male	9 female, 7 male	0 female, 5 male
**Age at examination (years)** (mean ± SD)	63.5 ± 10.6	66.38 ± 10.23	55.25 ± 11.54
**Amount of examinations**	45	44	5
**Daily wearing time (h)** (mean ± SD)	13.24 ± 3.58	8.83 ± 4.18	9.5 ± 2.87

**Table 2:** Summary of MRI safety of hearing implant systems by the manufacturer and distribution of the survey subjects; adapted from https://www.uniklinikum-dresden.de/de/das-klinikum/kliniken-polikliniken-institute/scic/downloads/mrtbroschuere.pdf (accessed on 20 June 2023) AND Leinung et al. [29].

Implant Type and Manufacturer				1.5 T MRI		3 T MRI		Max SAR (W/kg)		Number of Ears	Dis- Location
				Bandage Mandatory	Magnet Removal	Bandage Mandatory	Magnet Removable	Head (1.5T/3T)	Body (1.5T/3T)		
**Advanced Bionics**	**CI**	CII		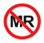						**x**	x
		C1.2								x	x
		C1.0								x	x
		HiRes90K (Advantage)		yes	yes	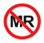		1.0/**x**	1.7/**x**	1	N/I
		HiRes Ultra		yes	yes	no	manda-tory	3.2/2.6	2.0/2.0	x	x
		HiRes Ultra 3D		no	yes	no	yes	3.2/2.6	2.0/2.0	x	x
**Oticon Medical**	**CI**	Neuro Zti		yes	yes	no	manda-tory	3.2/2.0	3.2/2.0	2	0
		Digisonic SP		yes	no	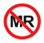		3.2/**x**	2.0/**x**	x	x
	**BCI**	BAHA Ponto		no		no			4.0/4.0	x	x
**Cochlear Ltd.**	**CI**	Nucleus Series CI24REH-R, CI422		yes	yes	no	manda-tory	1.0/1.0	1.0/0.5	0	0
		CI22M removable Magnet		no	manda-tory	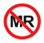		1.0/1.0	**x**/**x**	**x**	x
		Nucleus Profile Series	CI512	yes	yes	no	manda-tory	1.0/1.0	1.0/0.5	1	1
			CI522							1	0
			CI532							x	x
		Nucleus Profile Plus Series	CI612	no	yes	no	yes	2.0/1.0	1.0/0.5	x	x
			CI622							x	x
			CI632							x	x
	**BCI**	BAHA attract	BI400	no		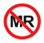		**x**/**x**	**x**/2.0	device not specified	**1**
		BAHA connect	BI300 BIA400 BIA300			no		**x**/**x**	3.2/2.0	1	
**MED-EL**	**CI**	C40, C40+		yes	no	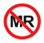		3.2/**x**	2.0/**x**	4	0
		PULSAR CI100								2	0
		SONATA TI100								4	0
		Mi1000 Concerto (PIN)								10	0
		Mi1200 Synchrony (PIN/ST)		no	yes	no	yes	3.2/1.6	2.0/1.0 *	33	0
		Mi1210 Synchrony ST								x	x
		Mi1250 Synchrony 2 (PIN)								3	0
	**MEI**	Vibrant Soundbridge VORP502		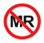						x	x
		Vibrant Soundbridge VORP503		no	no	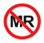		3.2/**x**	2.0/**x**	28	0
	**BCI**	Bonebridge BCI601		no ^#^	no ^#^	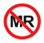		no limitation/**x**		2	0
		Bonebridge BCI602		no	no	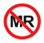		$/**x**	2.0/**x**	2	0

Cochlear Implant (CI), Bone conduction Implant (BCI), Middle Ear Implant (MEI), * for areas with <35 cm from cortex, otherwise 2.0 W/kg; ^#^ ≤1.5 T; $ only normal operation permitted; N/I no information provided.

## Data Availability

Not applicable.
